# Rabies in *Callithrix* sp. in the urban area of Niterói, Rio de Janeiro, Brazil

**DOI:** 10.1590/0037-8682-0402-2019

**Published:** 2020-03-16

**Authors:** Flavio Fernando Batista Moutinho, Marcela Garcia Araújo de Andrade, Viviane Moura Azevedo Nunes, Eduardo Cárdenas Nogueira Rubião, Helena Beatriz de Carvalho Ruthner Batista, Phyllis Catharina Romijn, Carlos Alberto Cattaneo, Fernando Guilherme de Oliveira, Rafael de Novaes Oliveira, Nairedisa Marcanth, Leilane Gorga Gaspar Ruas Silvestre, Fábio Villas Boas Borges, Sávio Freire Bruno

**Affiliations:** 1 Universidade Federal Fluminense, Faculdade de Veterinária, Niterói, RJ, Brasil.; 2 Fundação Municipal de Saúde, Centro de Controle de Zoonoses, Niterói, RJ, Brasil.; 3 Secretaria Municipal de Saúde, Instituto de Medicina Veterinária Jorge Vaitsman, Rio de Janeiro, RJ, Brasil.; 4 Pantharpia, Niterói, RJ, Brasil.; 5 Secretaria Estadual da Saúde, Instituto Pasteur, São Paulo, SP, Brasil.; 6 Empresa de Pesquisa Agropecuária do Estado do Rio de Janeiro, Niterói, RJ, Brasil.

**Keywords:** Primates, Lyssavirus, Rhabdoviridae

## Abstract

In Brazil, rabies occurs mainly within an urban cycle, in which dogs and bats are reservoirs. This paper aims to report the occurrence of rabies in *Callithrix* sp. in Niterói, Rio de Janeiro, Brazil. In June 2019 a hybrid specimen was referred for diagnosis. The Direct Fluorescent Antibody, Mouse Inoculation, and Polymerase Chain Reaction tests were positive. A phylogenetic analysis was compatible with antigenic variant 3, characteristic of *Desmodus rotundus*. New studies should be undertaken to elucidate the real role of callitrichids in the urban rabies cycle.

## INTRODUCTION

Rabies is a zoonosis that can affect all mammals and is caused by *Rabies lyssavirus* (RABV), genus *Lyssavirus*, family *Rhabdoviridae*. It has neurotropic characteristics that cause encephalitis, which is usually fatal. The disease occurs in two principal cycles, the wild and the urban, which may be interrelated. In the wild cycle, bats are the main reservoirs, and marmosets and wild canids are important RABV reservoirs in the Northeast region of Brazil. In the urban cycle, domestic dogs are the main reservoirs. The virus, present in the saliva of infected animals, is mainly transmitted by biting, licking, contact with injured skin or intact mucosa^1^. 

In recent years, the epidemiological profile of rabies in Brazil has changed, with the number of cases in primates and bats increasing, in urban areas and the wild, in contrast to a decrease in cases in dogs, cats and humans^2^.

This paper reports a case of rabies in *Callithrix* sp. (Primates, *Callitrichidae*) in the urban area of Niterói, Rio de Janeiro, Brazil.

## CASE REPORT

Niterói is a municipality within the Metropolitan Region of the State of Rio de Janeiro, which also includes the bordering municipalities of Maricá and São Gonçalo, as well as Guanabara Bay and the Atlantic Ocean. Occupying an area of 133,916 km^2^, it had an estimated population of 511,786 inhabitants in 2019^3^.

On 5^th^ June 2019, the Zoonosis Control Center collected a *Callithrix* sp. primate in the city centre, estimated to be six months old, displaying behavioural changes and signs of paralysis. After the animal died, the carcass was sent for rabies diagnosis at the Jorge Vaitsman Municipal Institute of Veterinary Medicine (IJV), Rio de Janeiro, RJ.

The results of the Direct Fluorescent Antibody Test and the Mouse Inoculation Test were positive. Brain fragments from the primate were then sent to the Pasteur Institute, in São Paulo, SP. The sample was identified with the number 2826/19 and subjected to RNA extraction and reverse transcription followed by polymerase chain reaction (RT-PCR), targeting the *N* gene encoding the RABV nucleoprotein: sense primer JW12 (5’-ACGCTTAACAACAARATCARAG-3’) and anti-sense primer P784 (5’-CCTCAAAGTTCTTGTGGAAGA-3’). The RT-PCR result was positive, so the amplicon was purified and subjected to genetic sequencing^4^. The genetic sequence obtained was edited using CHROMAS software (version 2.24 Copyright © 1998-2004 Technelysium Pty Ltd.).

For homology analysis, 514 base pairs of the RABV nucleoprotein were used, and 74 sequences retrieved from GenBank were included in the sample sequence 2826/19. These sequences were aligned with BioEdit software's CLUSTAL / W (version 7.1.3.0), and the phylogenetic tree was constructed using the Maximum Likelihood T92 G + I method with MEGA 7 and FIGTREE software.

**FIGURE 1:**
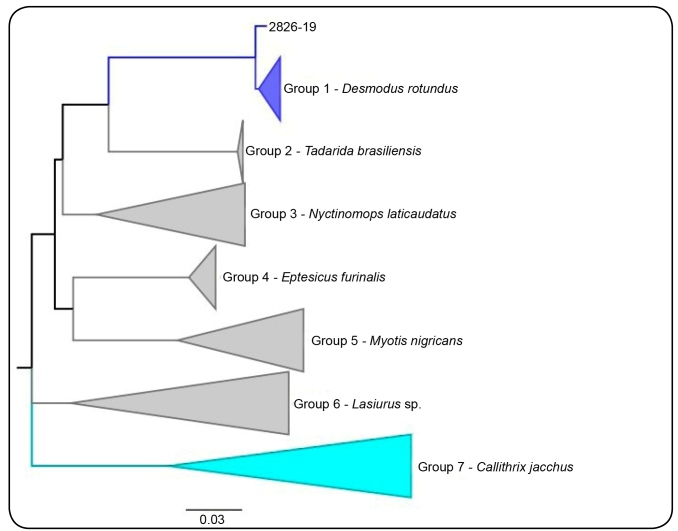
Phylogenetic tree built using the Maximum Likelihood method T92.

In the phylogenetic analysis it was possible to observe the formation of 7 groups: Group 1- RABV, with a genetic lineage characteristic of hematophagous bats *Desmodus rotundus*; Group 2- RABV, with a genetic lineage characteristic of non-hematophagous bats *Tadarida brasiliensis*; Group 3- RABV, with a genetic lineage characteristic of non-hematophagous bats *Nyctinomops laticaudatus*; Group 4- RABV, with a genetic lineage characteristic of non-hematophagous bats *Eptesicus furinalis*; Group 5- RABV, with a genetic lineage characteristic of non-hematophagous bats *Myotis nigricans*; Group 6- RABV, with a genetic lineage characteristic of non-hematophagous bats *Lasiurus* sp.; and Group 7- RABV, with a genetic lineage characteristic of non-human primates *Callithrix jacchus*. Sample 2826/19 was grouped in Group 1, with the other RABV sequences with genetic lineage characteristics of *Desmatus rotundus* hematophagous bats, compatible with antigenic variant 3 (Figure 1).

**FIGURE 2:**
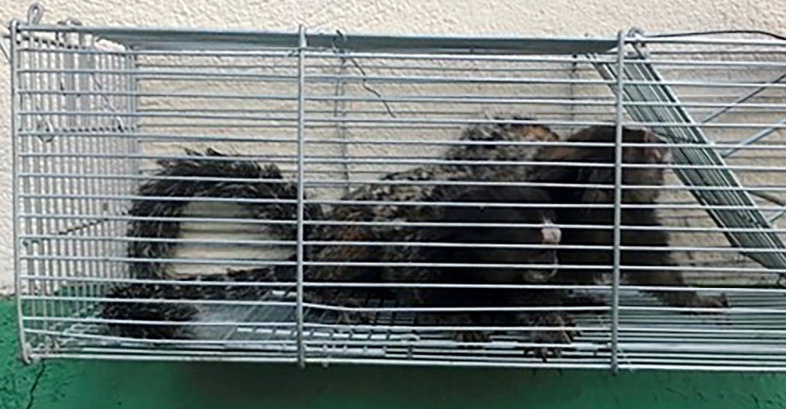
Captured marmosets (*Callithrix* sp) in a Tomahawk trap. (Source: Phoenix Environmental Projects).

At the site where the rabies-positive primate was found, three specimens of *Callithrix* sp. were captured using Tomahawk traps through the project “Monitoring of invading marmosets (*Callithrix* sp.) in the municipality of Niterói” by Phoenix Environmental Projects. Saliva samples collected from these primates using swabs were sent to the IJV laboratory and analysed using the Direct Fluorescent Antibody Test. All the samples were negative (Figure 2).

The Zoonosis Control Center then carried out prevention and focus control actions involving a transmission-blocking rabies vaccine in dogs and cats and disseminated health education to the population.

## DISCUSSION

Although rabies cases^5^ in primates in Northeastern Brazil are uncommon, there is an endemic transmission cycle involving *Callithrix jacchus*, with transmission to humans. In this cycle, unlike the one in Niterói, a virus variant was identified with no genetic relationship or antigenic proximity with the variants already recorded in the Americas for terrestrial mammals and bats^6^ ^,^ ^7^. However, Kobayashi et al.^8^ identified a case of rabies in a tufted capuchin monkey (*Cebus apella*) in the state of Mato Grosso, in 2010, that was also related to the bats’ variant, as in the case in Niterói.

The primate collected by the Zoonosis Control Center of Niterói presented progressive paralysis, which is one of the characteristic symptoms of paralytic rabies in primates^1^. However, in cases in the Northeast, some callitrichids had furious rabies^7^.

Regarding Niterói, Moutinho et al^9^. had already warned that urban rabies cases could occur due to several determinants and conditioning factors, including the presence of primates in urban areas. Since 2013, evidence of viral circulation in non-hematophagous bats has been present in the municipality.

Some wild animals, considered to be reservoirs of the rabies virus, such as bats and primates, have been developing synanthropic characteristics due to factors such as habitat destruction and the abundant availability of food in urban areas^10^.

The occurrence of rabies in *Callithrix* sp. is a matter of concern as these animals can approach human habitations and transmit the disease to domestic animals and humans. Additionally, many people feed these animals and keep them as pets. It is important to highlight that the species *C. jacchus* and *C. penicilatta*, native to the Northeast and Midwest regions, respectively, are considered invasive exotic species in the State of Rio de Janeiro^11^. The crossing of these callitrichids resulted in fertile hybrids of high plasticity that have adapted to natural and urban environments. Moreover, there is no abundance of predators to control their population. Kotait et al.^10^ highlighted, among the reservoir factors that favour the perpetuation of the rabies virus, high population density, the intensity of social interactions, and high displacement capacity. All these factors are present in relation to callitrichids in the municipality of Niterói.

All the cases of rabies diagnosed in chiropterans in the municipality involved the genus *Artibeus*, bats that use trees as shelter and are commonly found in urban areas. Additionally, because they often share trees with *Desmodus rotundus*, they may play an important role in rabies transmission^12^. This is one of the possible hypotheses that could explain the occurrence of rabies virus variant 3. Moreover, *Desmodus rotundus* colonies exist in the urban area of the municipality, and there have been several reports in recent years by the Zoonosis Control Center of cases of animal spoliation and one case of human spoliation^13^.

There is a shortage of immunobiologicals in Brazil, which is of concern as the vaccination of dogs and cats is an integral part of urban rabies control^1^. Moreover, the lack of immunobiologicals, both the serum and vaccine, for human use, makes post-exposure treatment difficult, leaving individuals vulnerable to the disease. Between 2012 and 2016, Niterói had an annual incidence of 37.2 x 10,000 inhabitants of human antirabies cases^9^.

Additionally, although the municipality has been achieving vaccination coverage of domestic animals that exceed state levels, the levels are below those recommended by the Ministry of Health^9^. In addition to this, many stray dogs and cats have not been vaccinated.

In this context, the educational actions developed by the Zoonosis Control Center, following the recommendations of the World Organization for Animal Health (OIE)^1^, are essential to make the population aware of the importance of vaccinating domestic animals and avoiding contact with wild animals, especially bats and primates.

The presence of callitrichids in urban areas could become a significant problem in controlling animal rabies, especially in places where there are exotic hybrids. This is because the urban environment and the lack of natural predators results in a high population density of callitrichids, which are possible reservoirs of RABV. The situation is exacerbated by people trying to touch and feed these animals or keep them in captivity.

Efforts should be made by the local government to prevent the occurrence of new rabies cases in the municipality. Research should be carried out to elucidate the precise role of these primates in the epidemiology of urban rabies.
